# Editorial: Endophytes and Their Biotechnological Applications

**DOI:** 10.3389/fbioe.2021.795174

**Published:** 2021-12-02

**Authors:** Adeline Su Yien Ting, Priscila Chaverri, Ru Angelie Edrada-Ebel

**Affiliations:** ^1^ School of Science, Monash University Malaysia, Malaysia, Malaysia; ^2^ Universidad de Costa Rica, San Jose, Costa Rica; ^3^ University of Strathclyde, Glasgow, United Kingdom

**Keywords:** biodiversity, bioactivity, biotechnology, endophytes, genome approach, green chemistry

Endophytes are microorganisms that are found within plants. They inhabit and colonize plant tissues without causing any visible symptoms (Petrini, 1991). The association of endophytes with their host plants have been shown to be beneficial, notably with improved tolerance of host plants towards biotic and abiotic stresses (Sun et al., 2010; Kumar et al., 2011). In the early years, research on endophytes were primarily on the valuable compounds produced, which have antimicrobial (Wang et al., 2014), antioxidant (Pan et al., 2017), or antitumor properties (Palem et al., 2015). These were meant for the agricultural, medicinal, and pharmaceutical use. In recent years, the function of endophytes to biosynthesize, biotransform, biocatalyze and biodegrade compounds, have become increasingly important. This led to their role in green chemistry, as endophytes can be used to generate products or biodegrade wastes in a more environmentally-friendly manner. In addition, the enzymes and pigments produced by endophytes are valuable as well, with applications extending to bioindustries and food applications (Castro et al., 2014; Dufosse et al., 2014). There is therefore, tremendous potential in biosourcing endophytes for various biotechnological applications.

The extensive research carried out on endophytes in the last few decades has improved our understanding of endophytes tremendously. This has led to many interesting endophytic isolates identified from a plethora of species found ubiquitously in nature, with their beneficial properties established (Ting, 2021). A typical biosourcing approach includes isolation, identification, bioguided assays, targeted developments of elucidated compounds, and downstream development to upscale their production. This approach has worked well in many studies, generating results for many of the existing literatures. However, these current practices are relatively laborious and are limited by sample size and bioassay techniques. Integration of new, precise and efficient biotechnological approaches has now become a necessity to facilitate biosourcing efforts.

With the upsurge in biotechnological breakthroughs in recent years, new approaches are integrated into endophyte research. As a result, most endophyte research would incorporate molecular typing for identification, and their bioactive compounds characterized and elucidated for further exploration using the omics approach or gene prediction. The benefit of these approaches is that the endophyte species and their multi-beneficial properties can be predicted via genetic information and annotation without bioassays. This allows for a more complete effort in biosourcing endophytes and bioprospecting their compounds and metabolites. The integration of biotechnological approaches expounded the discovery of endophytes for more significant applications.

The present research topic has 8 important articles; two review articles and six original articles, covering the main aspects of endophytic research. There are collectively three articles focussing on the role of endophytes in disease control, and one article on their role as plant growth-promoters. Two review articles on role and function of endophytes and their secondary metabolites and green chemistry. And another two interesting articles on the nature and behaviour of endophytes when present in plants.

The three original articles on role of endophytes in disease control were from Munoz-Guerrero et al., O'Sullivan et al. and Talukdar et al.. Munoz-Guerrero et al. reported on the bioactivity of 15 endophytic morphospecies for the control of anthracnose disease in Tahiti Lime flowers, a Citrus plant. Their bioguided assay revealed *Trichoderma atroviride* and *Xylaria adscendens* as effective biocontrol agents to control anthracnose incidence in Tahiti Lime flowers. O'Sullivan et al. adopted a similar bioguided approach to test 53 actinobacterial endophytes for their effectiveness in suppressing *Fusarium pseudograminearum* incidence in wheat. Talukdar et al. identified a tyrosol-producing endophyte (Collet*otrichum coccodes*) with antimicrobial activities strongly ascribed to the tyrosol produced. Tyrosol, a phenylethanoid, is an enzyme inhibitor with strong affinity towards bacterial tyrosol tRNA synthetase and fungal CYP45014α-lanosterol demethylase. The enzyme inhibition mechanism by tyrosol was further validated via molecular docking approach.

Aside from biocontrol activities, endophytes have growth-promoting attributes as well. Although this has been widely established, Chaudhary et al. took a different approach by integrating Rapid Annotation using Subsystem Technology (RAST) analysis to identify key genes responsible for growth promotion. In their study, the isolate *Rhizobium pusense* MB-17a was established for its growth-promoting properties via bioassays, and these attributes were further complemented with functional genomic annotation. The annotated genome confirms and identifies traits that have been verified by bioassays and predicted genes that aid in stress tolerance and growth promotion.

The importance of secondary metabolites and their bioprospecting approaches was reviewed by Sagita et al. The review includes the current state and future directions of genetics and genomics of endophytic fungi for bioprospecting efforts. It emphasizes on the importance of discovering the genetic basis for the phenotypic observations, and to elucidate the biosynthetic pathways. The authors also capture the challenges of bioguided assays, and how this mainstream approach may not necessarily be encompassing as “hidden gems” may be missed. The authors proposed a more inclusive approach, using the OSMAC (One-Strain-Many-Compounds) and multivariate approach. Understanding the biosynthetic pathways would allow for rational engineering for industrial purposes.

In addition to endophytes as sources of metabolite, the function of endophytes in green chemistry was also reviewed in this Special Topic. Choudhary et al. reviewed the role of endophytes in mediating the biocatalysis and biotransformation of products/substances into enantiopure compounds with biotechnological relevance. Whole-cell biotransformations or cocktails provide an alternative for chemocatalyzed reactions that usually is associated with perilous and hazardous environmental footprints. The structural analogs and pharmaceutical intermediates produced are possible novel chemistries and can have multi-purpose use for pharmaceutical and agricultural applications. The new products developed from biocatalysis and biotransformation include high value products such as products for the aroma and perfume industry, or flavouring. These products mimic the plant compounds, and are alternatives that can be produced from endophytes without destruction of plants. The endophytes can be engineered for future use.

The next two articles discussed the nature and behaviour of endophytes from an interesting perspective. Mohamad Zin et al. proposed that genome reduction in a particular endophyte indicates specialization to endophytic lifestyle. They used *Kitasatospora* sp. to perform bioinformatics analysis with AntiSMASH and BiG-SCAPE to identify metabolic pathways and their gene clusters (BGCs). Their study revealed that some genes were over-represented while others were under-represented. And the under-represented genes were likely the ones resulting in small genome.

Yousaf et al. demonstrated that the behaviour of an endophyte associated with the host plant is influenced by the hormones present. The study was conducted using *Bipolaris* spp. and the influence of auxin overload led to the shift of endophytic nature of *Bipolaris* spp. into a biotrophic pathogen. *Bipolaris* spp., a growth-promoting endophyte, elevated the IAA levels in the plant and the increase in IAA levels subsequently interfered with the phytoalexins and the brassinosteroids in the plants. As a result, plant growth was inhibited. The i*n-silico* analysis carried out further validated that high IAA levels resulted in the downregulation of brassinosteroid (BR) signalling.

In summary, the articles in this research topic “Endophytes and Their Biotechnological Applications” explore the multi-use of endophytes, their recent findings and how studies modelled using endophytes, help bridge the gap in the current advances in endophytic research. The studies here benefited from effective integration of new approaches such as gene prediction, in-silico analysis, molecular docking, to advance the knowledge on endophyte research. For future explorations, we predict that more biotechnological advances would be integrated, so that a more exhaustive data can be collected on the true potential of endophytes and their applications. These information would be invaluable for bioengineering purposes and future bioprospecting. Finally, our thanks go to the authors who contributed to the research topic, for sharing with us their insights and findings on endophytes.
